# ACCEPTABILITY AND FEASIBILITY OF A CAREGIVER FINANCIAL PREPAREDNESS PROGRAM

**DOI:** 10.1093/geroni/igac059.1841

**Published:** 2022-12-20

**Authors:** Lauren Stratton, Claire Grant, Monica Moreno, Kerry Lanigan, Katherine Judge, Sam Fazio

**Affiliations:** Alzheimer's Association, Chicago, Illinois, United States; Cleveland State University, Cleveland Heights, Ohio, United States; Alzheimer's Association, Chicago, Illinois, United States; Alzheimer's Association, Chicago, Illinois, United States; Cleveland State University, Cleveland, Ohio, United States; Alzheimer's Association, Chicago, Illinois, United States

## Abstract

Caregivers are often financially impacted, through health care costs, long-term care costs, and decreased income. A financial literacy program, Managing Money: A Caregiver’s Guide to Finances, was developed to provide information on financial impacts in caregiving. The virtual program was delivered synchronously with a live presenter through an online platform and was rated with high acceptability. The virtual program was then shifted to an online format. Changes included removing the live presenter and group format in favor of a voiceover and self-paced movement, the addition of videos, and online resources. The asynchronous, self-paced online delivery of the program was evaluated through a survey of 146 caregivers. Participants had a mean age of 45.85 (SD=5.65); 55% female; 75% White; 46% cared for their grandparent and 45% to their parent. A Likert scale was utilized to indicate satisfaction with the program (1=strongly disagree, 5=strongly agree). Overall, participants indicated that the program provided important information on managing money (M=3.97, SD=.83); helped to understand the content (M=3.89, SD=.91); was easy to read and understand (M=3.99, SD=.90); and would recommend the program (M=3.91, SD=.84). The explanation of content areas were rated on a Likert scale (1=poor, 4=excellent); participants rated areas including having conversations about finances (M=3.05, SD=.80); avoiding financial fraud and abuse (M=2.98, SD=.81); covering care costs (M=2.98, SD=.81); and organizing legal plans (M=3.09, SD=.80). Participants reported no difficulties that needed to be resolved when moving through the program. Overall, both the virtual and online formats were acceptable and feasible to caregiving populations.

